# Socio-Cultural Factors and Experience of Chronic Low Back Pain: a Spanish and Brazilian Patients’ Perspective. A Qualitative Study

**DOI:** 10.1371/journal.pone.0159554

**Published:** 2016-07-19

**Authors:** Daiana Priscila Rodrigues-de-Souza, Domingo Palacios-Ceña, Lourdes Moro-Gutiérrez, Paula Rezende Camargo, Tania Fátima Salvini, Francisco Alburquerque-Sendín

**Affiliations:** 1 Department of Physical Therapy, Catholic University of Avila, Avila, Spain; 2 Department of Physical Therapy, Occupational Therapy, Rehabilitation and Physical Medicine, Rey Juan Carlos University, Alcorcón, Madrid, Spain; 3 Department of Social Psychology and Anthropology, University of Salamanca, Salamanca, Spain; 4 Department of Physical Therapy, Federal University of Sao Carlos, São Paulo, Brazil; 5 Biomedical Research Institute of Salamanca (IBSAL), Department of Nursing and Physical Therapy, University of Salamanca, Salamanca, Spain; University of Manchester, UNITED KINGDOM

## Abstract

**Background:**

Low back pain (LBP) could be influenced by socio-cultural factors. Pain narratives are important to understand the influence of environment on patients with chronic LBP. There are few studies that have explored the experience of patients with chronic LBP in different socio-cultural environments. The aim of this study was to describe the experience of patients with chronic LBP in Spain and Brazil.

**Methods:**

A qualitative phenomenology approach was implemented. Chronic LBP patients from the University Hospital of Salamanca (Spain), and/or Federal University of São Carlos (Brazil) were included, using purposeful sampling. Data were collected from 22 Spanish and 26 Brazilian patients during in-depth interviews and using researchers’ field notes and patients' personal diaries and letters. A thematic analysis was performed and the guidelines for reporting qualitative research were applied.

**Results:**

Forty-eight patients with a mean age of 50.7 years (SD: ± 13.1 years) were included in the study. The themes identified included: a) ways of perceiving and expressing pain—the participants focused constantly on their pain and anything outside it was considered secondary; b) the socio-familial environment as a modulator of pain—most participants stated that no one was able to understand the pain they were experiencing; c) religion as a modulator of pain—all Brazilian patients stated that religious belief affected the experience of pain; and d) socio-economic and educational status as a modulator of pain—the study reported that economic factors influenced the experience of pain.

**Conclusions:**

The influences of LBP can be determined based on the how a patient defines pain. Religion can be considered as a possible mechanism for patients to manage pain and as a form of solace.

## Introduction

Low back pain (LBP) is a significant health care problem, where the 1-year prevalence for LBP ranges from 22% to 65% [1]. The economic burden of LBP involves high annual compensation costs, [2] and the impact of LBP on individuals can lead to a loss of health status in the form of loss of function and disability [3]. There is also evidence that LBP generally coexists with other medical conditions, such as depression and anxiety [4].

In Brazil, during 2002–2010, the 3-month prevalence of chronic LBP increased from 4.2% to 9.6%, which was partly due to an increase in the country’s life expectancy [5]. In Spain, the prevalence of LBP is around 20% and has remained unvaried during the last 5-year period [6]. In both countries, LBP has been associated with women, increasing age, and the lack of physical activity [5, 7].

Several social factors may also have an influence on LBP, such as earnings, ethnicity, access to health care [8] and low education levels [9]. Additionally, biomechanical factors, such as working in uncomfortable postures can also contribute to LBP [5]; all of these factors increase pain and disability [10]. Furthermore, low social support for chronic LBP is associated with a decrease in family support for the patient, the modification of family roles, partner dissatisfaction and frustration [3, 11]. Culture [12, 13], attitudes [14–16], beliefs and religion [17, 18] also seem to play an important role in LBP. Finally, social and cultural factors may be important in functional recovery as they affect the decisions made by the patients. How patients choose to cope with LBP [8, 13] can lead health professionals to construct erroneous stereotypes regarding certain minorities with chronic LBP [19], which could lead to discrepancies in diagnosis and treatment [20].

Designing studies that reflect the perspective of a patient with LBP would considerable help in understanding the suffering associated with the disease [14], and the implications it has on the patient’s life [21–23]. Moreover, these studies would assist professionals in the decision-making process to treat LBP [24–26], and in understanding the patient’s expectations regarding their treatment [4, 27–29], and their respective recovery [16, 30–31]. Also, the effect of disability associated with LBP [32], and the relevance of factors, such as culture, beliefs and the patients’ daily social environment could be ascertained [3, 12, 19].

In 2011, the Global Burden Study [32] reported that LBP was ranked as the greatest contributor to global disability in Western Europe and South America (measured in the number of years lived with disability). Although both regions ranked N° 1 in the study, presenting similar values, the meaning of LBP varied among the locations.

The description and comparison of the different perspectives of patients with LBP, either of Spanish [33] or Brazilian origin [34], would allow to understand the importance of different cultural [35] and social [36] backgrounds, and the motives that condition the search for help and treatment adherence or abandonment, to be analyzed [12, 37]. Consequently, the aim of this study was to describe the experiences and socio-cultural factors associated with the LBP of Spanish and Brazilian patients.

## Materials and Methods

### Study Design

A qualitative phenomenology study addressing the experiences of Spanish and Brazilian chronic LBP patients was conducted [38]. Qualitative studies are used to gain a deeper understanding of, and find explanations for, people’s behavior under specific circumstances, such as disease [39]. Furthermore, qualitative studies have been used to study people with chronic pain and pain self-management [40]. The main characteristic of this method is that researchers become closely involved in data acquisition and analysis. Data collection requires researchers to interact with the study participants and their social context, which allows some degree of mutual influence [38]. In the field of qualitative studies, phenomenology attempts to understand how individuals construct their world view, and looks through a window into other people's experiences. Such experiences will always have meaning for the people who have lived them, and therefore qualitative phenomenology design utilizes first-person narratives from patients [38].

Previous studies [4, 21–22, 41] have supported the relevance of pain narratives. Webster and Harden [41] reported that: “(…) the human effects of real-world pain that go beyond a pain intensity (…) the pain experience is more than a transaction of neurotransmitters with action potentials to be blocked or modulated (…) Life´s experiences, aspirations, and emotional needs are essential considerations to treating the whole person.” (p.1811). Also, Stisen et al. [4] showed that it is important to include the deeper meaning of metaphors and the personal story behind the expression of pain as a way of understanding each individual with LBP.

### Participants

To be eligible for the study, participants had to suffer from chronic non-specific LBP diagnosed by an Orthopaedic physician. LBP was defined as pain between the costal margins and the inferior gluteal folds, which is usually accompanied by painful limitation of movement and may be associated with referred pain running down the leg [32]. Also, patients showed moderate disability measured by Oswestry Disability Index (ODI) [42, 43], and had to participate in a follow-up program at the University Hospital of Salamanca (Spain), and/or the Federal University of São Carlos (Brazil), for Spanish and Brazilian patients respectively. The objective of the program was to evaluate, treat and follow up, in conjunction with Brazil, patients diagnosed with LBP living within a community and moderate disability (ODI score: 20% to 40%) [44], living within a community. This subject profile reflects the typical course of LBP, which tends to stabilize over time in a moderate disability status [45, 46]. The program was established in collaboration with the University of Salamanca and the Federal University of São Carlos, and included the study of the evolution and impact of LBP on the lives of the patients involved. All participants read and signed an informed consent form. Exclusion criteria were established as: a) younger than 18 years old; b) pain of infectious or oncologic origin; c) comprehension difficulties during the interview and d) refusal to participate in the study.

### Sampling strategies

A purposeful sampling strategy was employed and can be defined as the selection of individuals based on purposes associated with addressing a specific aim of the research study [38, 47]. It involves deliberately selecting participants, in this case LBP patients, to provide relevant information regarding their experience with LBP [38]. As previously commented, the recruitment criteria were defined to select patients who lived within a community and to describe the impact of pain in their lives into their own social environment. Thus, hospitalized or ambulatory clinic patients were not included in the study. In our study, this strategy was used to gather information from the LBP patients themselves [38, 47]. Among the different types of purposeful sampling techniques, the sequential sampling method was employed with the aim to access a substantial range of experiences that enabled comparisons to be made. This type of sampling was designed to reflect the context and experiences of a particular group, in this case the LBP patients [38]. The patients were included progressively as they accepted to participate in the study. The enrollment of the patients was not subjected to any probabilistic method (randomization). Finally, forty-eight patients were included within the sample (22 Spanish and 26 Brazilian patients) and none withdrew from the study. The researchers made initial contact with the patients through the Medical Chief in each of the Brazilian and Spanish Orthopaedic Medical Services. The researchers explained the purpose and design of the study to the patients during an initial face-to-face contact session. A 1-week period was then allowed for patients to decide whether or not they wished to participate. During the second face-to-face session, they were asked to give written informed consent and permission to tape the interviews if they wished to participate in the study. Following this, data were collected and the interviews were completed.

### Researchers´ backgrounds and procedure

The research team consisted of six members (4 women and 2 men), three from Brazil (DPRS, PRC, TFS) and three from Spain (DPC, LMG, FAS). Each group was led by an expert researcher with experience in qualitative designs (DPRS, DPC). The other members of the research team were working at one of the respective university (PRC, TFS, LMG, FAS). At the time the study was carried out, all of the researchers had their PhDs, except one member who had a Master’s Degree (DPRS). Four members were physiotherapists (DPRS, PRC, TFS, and FAS), one psychologist (LMG) and one nurse (DPC). Previous to working at the University level, 4 members of the team had clinical experience in rehabilitation and pain (DPRS, PRC, TFS, and FAS), one member in neurology (DPC) and one in psychology (LMG). In addition, all members of the team had research experience in chronic musculoskeletal pain. The authors had no previous contact with any of the participants.

The teams from both countries met once a week via teleconference. The contents of the meeting were related to: quality control criteria, methodological aspects for data collection and analyses, and updates regarding the progress of the study. The meetings were conducted in both languages, i.e. Spanish and Portuguese.

### Data collection

Data were acquired over a one-year period from January 2013 until December 2013. The methodology used for data acquisition is outlined in Table 1.

**Table 1:** Data collection process.

**Data collection**		
**Type of interview**	**Documents**	**Number of interviews**
Unstructured	Researchers’ field notes from 48 participants + 8 Diary fragments + 18 personal letters	10 Brazilian patients 10 Spanish patients
Semi-structured + guide questions		16 Brazilian patients 12 Spanish patients

The data collection process began with an in-depth unstructured interview [48], always beginning with the following question: “What is your experience with LBP?” The in-depth unstructured interview consisted of direct but open questions that allowed the patients to share their personal experiences. The aim of this first question was to seek potential themes and topics that could be further expanded. Subsequently, a semi-structured interview based on a question guide was performed (Table 2). The aim here was to elicit further information regarding the specific themes and topics of interest that had emerged from the unstructured interview [48]. The question guide was developed after reviewing the patients’ accounts obtained during data collection.

**Table 2:** Question guide for the semi-structured interview.

**Research topics**	**Questions asked**
The meaning of pain	What is your experience with Low Back Pain? Regarding living with pain, can you tell me what the most relevant aspect of this is for you in particular?
Daily activities	How does pain affect your daily life?
Social relations	How does pain affect your relations with your friends and other people in your neighborhood?
Family	How does pain affect your relationship with your partner and family?
Strategies for controlling pain	Have you used or are you currently using any strategies to control or decrease your pain?
Factors that influence/modulate pain	Which factors or elements do you think might influence or modulate your pain?

The interviews were tape-recorded and transcribed verbatim. Personal documents (diary fragments and personal letters) provided by the patients and the researchers’ field notes were collected. The personal data collected from patients provided a rich source of information since they describe personal experiences from the patients’ point of view [38].

During the interview, the researchers made notes, including a description of the environment, patient non-verbal responses to questions, the use of metaphors in their narratives, and other relevant points emerging in the interview.

A total of 48 interviews involving 48 patients were conducted. These interviews comprised a total recording time of: 1680 minutes (28 hours) and 1335 minutes (22.25 hours), for Brazil and Spain respectively. Also, the median duration of an interview was 58 minutes, with a range of 37–102 minutes, for Brazil, and 44 minutes, with a range of 32–100 minutes for Spain. Eighteen personal letters and 8 diary entries were collected from the patients, together with the researchers’ field notes from the 48 participants. All interviews were conducted in a separate office at the Hospital and University facilities. Sociodemographic data were obtained following the interviews and were provided independently by the participants in a self-completed questionnaire.

### Data analysis

A complete and literal transcription of each of the interviews, researchers’ field notes and patient’s documents were drafted. The texts were collated to allow qualitative analysis to be performed [38]. In order to include the personal documents into the analysis they were first check for authenticity (generated within the context of LBP), credibility (produced by the actual LPB patients), representativeness (described personal experiences) and meaning (provided information relevant to the study) [49]. Each researcher was responsible for data collection, treatment and analysis. A thematic analysis was performed separately [50] by the researchers of each country. This process began by highlighting the most descriptive content to obtain meaningful units, and then delved deeper to reduce and identify the most common meaningful groups. Thus, groupings of meaningful units were formed, referring to similar points or content that allowed the topics that described the receivers’ experience to emerge. The thematic analysis was carried out separately on the interviews and using the personal documents. In those patients that had been interviewed and provided personal documents the obtained results were integrated separately in the same analysis matrix. The results of the analysis were subsequently combined in joint meetings, where the data collection and analysis procedures were discussed. In the case of differences in opinion, theme identification was performed by consensus among the team members. Following on, the entire research team held joint meetings to show and combine the results of the analysis from each country. At that point, all researchers analysed and discussed the Spanish and Brazilian results, and identified final themes by consensus among all members. All of the researchers participated in the coding and analysis, but the task was overseen by the researchers with experience in qualitative research (DPC, DPRS). No data analysis software was used.

### Quality Criteria

The guidelines for conducting qualitative studies established by the consolidated criteria for reporting qualitative research [51] were followed (http://www.equator-network.org/). Data reliability method [52] consisted of: a) cross-triangulation by the research team, which included a plan of meetings where the cases analysed by each team member were presented to obtain a consensus; b) auditing the material obtained from 16 randomly selected cases by an external researcher; and c) patient verification. Patient verification was carried out in two steps: 1) post-interview: the tape-recorded interview was presented to the patient to validate the contents and allow for additional information to be added and post-analysis; 2) analysis resulting from the interview: the units of meaning and the groupings of meaning identified by the researcher were presented to the patient to confirm that the results of the analysis actually reflected the patient’s perspective.

The quality control that was applied to the interview transcription was taken into special consideration. In the first phase, the interviews were transcribed using the language of the country of origin so that the researchers from that country could carry out the corresponding analysis. Afterwards, all transcriptions together with the analysis conducted (qualitative data and results) were translated (from Spanish to Portuguese and from Portuguese to Spanish) and sent to researchers of each country (Brazil to Spain and Spain to Brazil) in order to prepare for the joint meetings. Collecting qualitative data in one language and presenting the findings in another language forced the researchers to take translation–related decisions that had a direct impact on the trustworthiness of the research and its findings [53]. The translation procedure used in the present study included the following stages: a) First, all verbatim transcription of the contents of the interview data into Spanish and Portuguese (and vice versa), followed by content analysis, b) second, concepts and themes emerged after the verbatim transcriptions and performance of the analysis. Two bilingual translators translated all qualitative data and results into Spanish and Portuguese (and vice versa). The final Spanish and Portuguese versions were finalized by the agreement between both translators, c) then, bilingual staff back translated the transcriptions, qualitative data analysis and results into the original language. Back translation involves translating from the target language back to the source language in order to evaluate the equivalence between the source and target versions [54] and d) finally, a conceptual equivalent regarding the words used was achieved by selecting those which most native speakers could understand. An expert panel of researchers and translators was involved in reaching a final agreement of the translations used in this work [54].

### Ethics

This study was approved by the Clinical Research Ethics Committee at the University of Salamanca (project number: 7-2-12). It was performed in accordance with the Declaration of Helsinki. Permission to record the interviews was always sought prior to their being performed. Written informed consent was obtained beforehand.

## Results

Forty-eight LBP patients with a mean age of 50.7 years (SD: ± 13.1 years) were included within the study. The median of the age of the LBP patients was 47 years (range 38–65). Table 3 shows the details of the sociodemographic data of the 48 patients.

**Table 3:** Sociodemographic data.

**Data collection**		**Spanish participants n = 22**	**Brazilian participants n = 26**
**Clinical characteristics**	Time suffering pain (months)	115.6±96.9 (89.1–154.6)	116.7±90.2 (82.5–153.6)
	Current pain intensity (NPRS, 0–10)	6.6±2.4 (5.7–7.5)	7.0±1.5 (6.4–7.8)
	Treatment 1 (NSAIDs)	14	16
	Treatment 2 (physical therapy)	11	11
	Disability (ODI)	28.4±15.3 (22.4–36.2)	31.3±16.2 (24.5–37.1)
**Age (years)**		52.2 ± 14.7	49.3 ± 11.7
**Sex**	Male	12 (54.5%)	15 (57.7%)
	Female	10 (45.4%)	11 (42.3%)
**Marital status**	Married	15 (68.2%)	14 (53.8%)
	Single	7 (31.8%)	11 (42.3%)
	Widow	0 (0%)	1 (3.8%)
**Number of children**		1.7 ± 1.5	2.0 ± 1.4
**Job**	Student	1 (4.5%)	0 (0%)
	Retired	4 (18.2%)	2 (7.7%)
	Employed	17 (77.3%)	24 (92.3%)
**Educational level**	No basic studies	1 (4.6%)	3 (11.5%)
	Studies equivalent to High School or less	15 (68.2%)	21 (80.8%)
	University Degree holder	6 (27.3%)	2 (7.7%)
**Residence**	Rural setting	10 (45.4%)	1 (3.8%)
	Urban setting	12 (54.5%)	25 (96.2%)
Social class*	Upper	0 (0%)	1 (3.8%)
	Middle	19 (86.4%)	20 (76.9%)
	Lower	3 (13.6%)	5 (19.2%)
	Poor	0 (0%)	0 (0%)
**Number of people patient lives with**		2.4 ± 1.5	3.1 ± 0.9
**Religion**	Believer	16 (72.7%)	26 (100%)
	Non-believer	6 (27.3%)	0 (0%)

Quantitative data are expressed as Mean ± SD (95% Confidence interval). NPRS: Numerical Pain Rating Scale; NSAIDs: Nonsteroidal Anti-inflammatory Drugs; ODI: Oswestry Disability Index; SD: standard deviation

* Social class was classified in: Upper class: Executives of high level management and companies of approximately 10 employees, professionals holding higher Degrees and technicians. Artists and sportspersons. Middle class: Administrative employees and professionals in administration and business management. Personal service and security workers. Self-employed. Supervisors and skilled workers. Lower class: Semiskilled and unskilled manual workers. Poor class: Other types of untrained and inexperienced workers.

The themes representing the experiences of patients of LBP were extracted from the interviews. Four specific themes emerged from the material analyzed: a) ways of perceiving and expressing pain; b) socio-familial environment as a modulator of pain; c) religion as a modulator of pain, and d) socio-economic and education status as a modulator of pain. In S1 Appendix, we included the narratives taken directly from the interviews, diaries and personal letters regarding the four themes identified within the study.

### Ways of perceiving and expressing pain

Regarding the experience of pain, most participants, from both countries, reported that pain had become “everything”; it was seen as a presence in itself that had in some way “taken over” the patient’s body. The participants focused constantly on their pain and anything outside it was considered secondary. This type of pain affects all levels of life: physical, psychic and social. Similarly, all patients perceived that any physical disturbance, pain, or worry seemed to take priority over everything else, and their whole lives were directed towards avoiding the appearance or any increase in their pain.

On describing and expressing LBP, the Spanish patients described it as an isolated phenomenon, using specific and aggressive terms (*like being stabbed with a knife*, *it eats you up*, *it is rabid*, *a deep wound*) whereas the Brazilian patients described it in relation to activities and circumstances where pain arises (work, housework…). Most of the Brazilian patients appeared to be more positive about their pain, even in cases in which owing to their clinical condition it was chronic and constant. They showed a cheerful, positive attitude and were aware that such an attitude would be beneficial to them.

Regarding the differences between the patients from the two countries, the way of expressing pain varied. Thus, the Brazilian patients claimed that *being cheerful and positive helps*, whereas the Spanish patients reported that *feeling sad and being negative* was not good for them.

### The socio-familial environment as a modulator of pain

All participants stated that pain was something “personal”; they lived it and felt that nobody was able to understand their experience. This led them to draw away from their immediate social environment. Moreover, most patients (from both countries) reported that their family environment demanded extra of them; they urged that the patient make an effort to overcome pain, as if “forgetting about it” were an option. The patients reported that sometimes they were even accused by family members of being exaggerated or malingering. The lack of a clear diagnosis in some cases made them feel less well understood. They sometimes even lived their pain as though it were a scourge.

In both countries, the patients’ narratives pointed to two perspectives in relation to their friendships. On the one hand there were participants who had lost friends as a result of the changes in their lifestyles brought about by the pain they were undergoing (they stopped going out, stopped travelling, etc.) and, on the other hand, there were individuals who struggled to keep their friends, avoiding changing their habits even though this was not to their benefit.

Regarding differences between the participants from the two countries, the reports of the Brazilian patients pointed to more support from friends and family, with few changes in their daily activities due to pain. By contrast, in the Spanish patients there were more reports revealing a change in their lifestyles, together with the use of more expressions referring to how lonely they felt, how they felt rejected, or how they were suspected of malingering by their family members.

### Religion as a modulator of pain

All the Brazilian patients stated that religious belief affected the experience of pain. For these patients, faith and belief was a relief, a constant and necessary support that gave them the strength to cope with their pain. No accounts of pain being perceived as divine retribution or anything similar were recorded.

Half of the Spanish patients stated that religion could affect pain, although with interpretations different from those of the Brazilian patients. For these Spanish patients, religion was mentioned in terms of its cognitive and mental effects in the perception of pain, where there is a rational explanation of the mechanism concerning the belief that faith helps patients. The other half of the Spanish patients did not believe that religion could help to mitigate lumbar pain, even though some of them were believers.

### Socio-economic and educational status as a moderator of pain

Nearly all the patients included in the study reported that the economic development of the country of origin influenced the experience of pain. Good health coverage and the existence of resources destined for the treatment of LBP were factors that reduced the experience of pain. Most of the Brazilian patients stated that *the rich* may suffer less because they have access to better health care, while *the poor* must suffer more owing to their economic limitations. In the case of the Spanish patients, the results were not as patent since they considered their own health system sufficiently universal to cover lumbar pain for all sectors of the population.

In both countries, living in urban or rural areas was also perceived as a factor that affects the experience of pain. Thus, some Brazilian and Spanish patients reported that living in a rural setting increased the likelihood of developing LBP. In the country, the possibilities of rest or stopping work are slim, and pain must be supported as long as this allows the individual to work.

Regarding the differences between the patients from both countries, most of the Brazilian patients described experience of more pain than the Spanish ones, but they were able to bear it better in their daily lives. Differently, the Spanish patients complained more and had difficulty in accepting and assimilating the pain.

Some patients, from both nationalities, stated that having more education decreases the perception of lumbar pain because better educated people will have better knowledge about how to seek solutions, and understanding of the mechanisms of pain. Some Brazilian patients related a low education level to having more menial jobs, which were more likely to produce lumbar pain. By contrast, the majority of Spanish patients stated that the level of education was not related to lumbar pain or the way of coping with it.

## Discussion

The results of this study show that for all patients pain had become the main axis of their lives. Spanish patients described pain in an aggressive manner, while the Brazilian patients’ attitude towards describing their pain was more positive. The patients manifested the difficulty in understanding their pain and recounted the conflicts that had arisen with family members. The Brazilian patients believed that having few resources and a low education level had an influence on pain. On the contrary, religion was perceived as a source of relief for these patients. Also, all of the patients related living in rural areas to more pain, as the work carried out was more manual.

Culturally-specific attitudes about the role and meaning of pain can influence the perception and response of patients to their own pain [21]. Thus, culture can in turn influence the request for treatments and care to relieve pain [13, 35]. In fact, different patients have different thresholds for seeking care or expectations about the care they receive, and they also hold beliefs that influence the adherence to treatment [23]. As a result, when patients seek care, they have certain beliefs about the cause of their symptoms, concerns about their illness, and expectations about treatment [14, 35].

Previous studies [3, 11, 19, 21] reported that lumbar pain interrupts patients’ lives and becomes the focus of attention, which limits their daily activities. LBP may generate fear-avoidance beliefs, which limits patients’ lives and has important social, familial and job-related repercussions [3, 14, 23, 55].

Our results reveal that the narratives of the Spanish patients are more aggressive and negative in comparison to the Brazilian’s. Additionally, previous studies have shown that the intensity and the duration of pain, and the associated disability have influence on how pain is expressed [55]. However, our results show that the intensity and duration of pain for both nationalities were similar. One possible explanation for this may be that, in some occasions, the Spanish patients with LBP feel guilty and tend to isolate themselves because they think they are a burden to others [33]. Darlow et al. [14] and Urquhart et al. [15] reported that beliefs about pain and the ways used to describe it (negative attitudes) may govern the perception of the intensity of the pain and disability. Furthermore, it has been reported that the sex of the patient and cultural aspects may drive the presentation and expression of pain in different ways, as demonstrated for Muslim women [12, 20] and aboriginal Australians with LBP [56]. Duggleby [57] reported that members of the Latin/Hispanic community express pain in a fatalistic way, similar to other cultural traits.

Our results also show that all patients from both countries feel pressure from their families and workplace, which is a result that coincides with previous studies carried out in Spain [33] and Brazil [34]. In fact, it has been reported that LBP produces inter-family and partner conflict [8, 11, 33, 34], a decrease in family support for the patient, and pressure on patients to return to work. Arcanjo and Silva [34], Lin et al. [19] and Tavafian et al. [12] reported that there are certain cultural obligations (at work, family and home care) that put pressure on patients with LBP. Likewise, the importance of the family in Latin/Hispanic cultures may modify the behavior and attitudes of patients with chronic pain [57] and this may affect their health and recovery [34]. In a similar way, Bailly et al. [3] and Cano et al [33] showed that LBP patients reported a negative self-perception in social interactions, felt misunderstood and unsupported, partly due to the absence of visible signs of the condition.

Our study reveals how Brazilian patients have a strong belief in the importance of religion in LBP. In Brazil, Arcanjo and Silva [34] reported how faith and religion can help in the acceptance of situations of great pain, and help to enhance the patient’s religious commitment. Also, Wachholtz and Pearce [18] described that religion may either help or impede the handling and experience of chronic pain. Other previous studies [18, 58] have shown that religion can serve to give meaning to pain, which helps to cope with and overcome it. Furthermore, Baetz and Bowen [59] report that people with faith who have LBP tend to suffer less from fatigue and chronic pain, and more likely to have greater psychological well-being. In fact, the patients with poorer health were more likely to participate in religious activities as a coping strategy [60]. In contrast, our results show that the Spanish patients do not believe that religion has influence on LBP. As a possible explanation, Spain has experienced progressive secularization during the last decade. This fact has put majority of the population away from the catholic religion, rituals associated with its practice, and promoted a switch towards a more civil and non-religious society [61].

Socio-economic and educational factors are also perceived as modulators of pain. The manifestation of pain is inextricably linked to those factors that make up the individual´s social environment, such as social network, literacy, and socioeconomic factors [21–22].

Furthermore, our results have exposed the differences between Brazilian and Spanish patients with respect to their opinion as to whether healthcare coverage can have influence on LBP. In Spain, Cano et al. [33] have showed how LBP patients intensely use the healthcare services offered by the national health system because they believe that they are better than those offered by the private sector. Meanwhile, in Brazil, the healthcare system, in some occasions, cannot attend to all rehabilitation needs of patients with LBP [34]. This can be explained by the existing differences between both countries with respect to access to healthcare. Spain has national healthcare model [62] that permits unlimited access to the majority of treatments and diagnostic tests for LBP, while in Brazil the *Sistema Único de Saúde* presents a mixed healthcare model, 75% public and 25% private [63], which can cause the rehabilitation of LBP to be limited [34, 64].

Moreover, Shaw et al. [8] described that low levels of education and low wages elicit a stronger experience of pain; recurring episodes increase in frequency, and the ailment becomes chronic and disabling. In fact, the association between low education levels and the presence of chronic pain due to musculoskeletal pathologies has been described in Spain [6, 7]. Education could help patients to become aware of their pathology and this may facilitate their adherence to treatment. Education also tends to lead people to adopt healthy life styles that will prevent risk factors for back pain, such as obesity and tobacco smoking. In contrast, Briggs et al. [65] reported that the beliefs and general behavior is more meaningful regarding disability by LBP than by the level of education.

Previous studies have demonstrated a similar prevalence of LBP in both rural and urban settings [8]. Nevertheless, Holmberg and Thelin [66] reported that self-employed patients in a rural setting had shorter recovery times and returned earlier to their jobs despite their LBP. In fact, it has been described that there may be job conflicts that harm the patient’s/worker’s image [67]. One possible explanation could be that the workers in rural areas exhibited high self-efficacy scores, positive perceptions about LBP, and stoical resilience and commitment to continue to work despite their LBP [68].

### Limitations

Although some authors recommend that interviews should be held alone [38, 46], 6 patients expressly requested that their partners also be present. In these cases it was found that the partners participated actively in the care of the patients, who delegated certain aspects of their care to their partners. Furthermore, during the interviews it was observed that far from having an inhibitory effect, the presence of the partners acted as an efficient catalyst and memory aid, and the partners were able to verify the patients’ accounts. Despite this, any possible "pollution" by the partners was controlled by preventing them from monopolizing the conversation during the interview and redirecting the interview when distractions arose due to the discussion of irrelevant aspects. Another limitation is that the great majority of the patients from both countries perceived themselves as being middle class. This can be explained by the type of job they had and how they consider that the type of job could be classified in one or another social category. Finally, the social and cultural differences between countries could modify the experiences narrated by the patients. To minimize this possibility, the program utilized to recruit the patients and the criteria that allow the patients to participate were identical for both countries. Likewise, at the time when data were collected and analyzed, periodic meetings were carried out between the members of the research team of both countries to ensure that the same methodology was applied during the study.

## Conclusions

The influences of LBP can be determined based on an understanding of the meaning given to pain by patients and the effect of socio-cultural factors. Our results can aid health professionals in the understanding of LBP in multiethnic and multicultural areas. Religion can be considered a possible instrument for the handling of pain in some patients who are believers, although further work is still required. In both Spain and Brazil, this work provides a first step towards increasing the awareness of the perceptions regarding the quality of life of LBP patients.

## Supporting Information

S1 Appendix Narratives from Low Back Pain patients. (DOC)
