# Supplementary material for: Socio-Cultural Factors and Experience of Chronic Low Back Pain: a Spanish and Brazilian Patients’ Perspective. A Qualitative Study
Source: PLoS One. 2016 Jul 19;11(7):e0159554. doi: 10.1371/journal.pone.0159554 (PMC4951039; doi:10.1371/journal.pone.0159554)
Supplement: S1 Appendix — (DOC) [file pone.0159554.s001.doc]

**S1 Appendix. Narratives from Low Back Pain patients.**

| **Themes** | **Patients´ Narratives** |
| --- | --- |
| **Ways of perceiving and expressing pain** | Focusing on pain: *“I now feel as if it is not me living this, something else is driving me, my pain. It has a life of its own: me, on one hand, and the pain, on the other….” (Sp15, 46 years old)*  Priorizing pain: *“I can’t avoid it, it affects me as a person, my body, my relations, how I relate to people, my partner… I worry about everything lest it trigger more pain…” (Sp9, 37 years old)*  Positive attitude: *“It hurts, of course it hurts; sometimes I can’t even get out of the house, although I still have things that make me happy: my life, my children, my family… “(Bp19, 28 years old)*  Feeling sad: *“…I can’t allow myself to feel sad. It’s a vicious circle. The more you think about it the sadder, the more negative you become and the less you feel like going out or doing things and so you get even sadder. You must stop feeling unhappy and thinking negative things” (Sp10, 33 years)* |
| **The socio-familial environment as a modulator of pain** | Living pain as scourge: *“Sometimes people look at me as though I wanted to have this pain, as though I could choose to have it or not have it. They don’t understand that having to live like this makes you feel different, not understood, marked… Now I understand why people want to be alone; at least like that you avoid suspicion and having to give explanations…” (Bp12, 63 years old)*  Loosing friends: *“I was always well in with my friends but now they have failed me. Because I can’t keep up with their pace, it seems as though I no longer belong to the group…” (Sp13, 67 year old, semi-structured interview)*  Keeping friends*: “Although it hurts and I know that the next day I will pay for it dearly, I go out with them. I have to struggle to keep up, but pain won’t stop me. If I don’t do this who will do it for me?” (Sp4, 44 years old)* |
| **Religion as a modulator of pain** | Relief trough belief: *“Despite all the pain, I keep on. God helps me and sustains me. He tells me that the pain can’t beat me down” (Bp8, 55 years old),* *“I am a practicing Catholic and I think that belief can be a good ally against pain… sometimes I get pain crises that make me very ill and then I pray… it strengthens me and gives me the strength I need for times like that…” (Bp2, 44 years old)*  Religion its cognitive and mental effects in the perception of pain: *“…I believe that the faithful manage to interpret their pain differently, they manage to convince themselves that their back isn’t going to hurt, or something is going to cure them; the only thing they are doing is benefitting from a placebo effect in their brains.” (Sp4, 28 years old)*  Religion could not help to mitigate lumbar pain *“I’m Catholic and I know many priests and when they are in pain it’s the same as for the rest of us. Yes, you might try to take it differently, that may be possible, but when it hurts, it hurts…” (Sp5, 37 years old)* |
| **Socio-economic and educational status as a moderator of pain** | For Brazilian patients health coverage reduced the experience of pain: *“It’s not that in Brazil we can stand pain better, it’s that we have to wait longer. If in other countries people don’t feel so much pain it’s because they have better access to health care and the doctors control the pain earlier and with resources for all, rich and poor…” (Bp5, 67 years old)*  For Spanish patients health coverage not affect the experience of pain: *“In Spain we don’t notice the influence of money so much. Yes, it may be better to have money, to go to a private clinic, but in theory we all have same access to pain-mitigating treatment”. (Sp18, 65 year old)*  For Brazilian patients good economic state reduced the experience of pain: *“People who have money can have a more continuous specialized treatment; they can hire physiotherapists and they get better sooner” (Bp3, 70 years old)*  Living pain in a rural setting: *“I think pain appears if you have a hard, physically intense, job, and working in the fields is the epitome of this. It’s very demanding; you have to be at it all day long and you can never take a holiday. Who else would look after your animals and the farm?” (Bp20, 67 years old)*  Putting up with pain: *“I’ll keep on working until I drop. My dad never missed work, he always just put up with it. He was responsible and had dignity, as it should be…” (Bp22, 70 years old)*  For Brazilian patients the educational level and menial jobs were related to LBP: *“… a person who has better studies has less pain that someone who has less education... that person has more experience and knows what to do and what not to do…” (Bp16, 45 years old), “…people who have more education have less pain because they do a different kind of job. I didn’t study; I went to work in a factory and worked my back off there. Education has clout. You won’t find a doctor, professor or dentist who does too much work and develops backache” (Bp15, 63 years old)*  For the Spanish patients the educational level was not related to LBP: *“It doesn’t matter; what’s important is how you deal with it. I have a doctor whose back is out of whack because it runs in the family. He’s always complaining. Does education help in all this? I don’t think so.” (Sp12, 61 years old)* |

Sp : Spanish patient; Bp; Brazilian patients. All patient´s narratives were followed by the number of participants and age.
